# Daily mapping of Australian Plague Locust abundance

**DOI:** 10.1038/s41598-020-73897-1

**Published:** 2020-10-09

**Authors:** Stéphane Mangeon, Allan Spessa, Edward Deveson, Ross Darnell, Darren J. Kriticos

**Affiliations:** 1 Commonwealth Scientific and Industrial Research Organisation, Data61, Brisbane, Australia; 2 Fenner School of Environment and Society, Australian National University, Linnaeus Way, Acton, ACT 2601 Australia; 3 Australian Plague Locust Commission, 50 Collie St, Fyshwick, ACT 2609 Australia; 4 Commonwealth Scientific and Industrial Research Organisation, Health and Biosecurity, Canberra, Australia; 5 School of Biological Sciences, University of Queensland, St. Lucia, QLD Australia

**Keywords:** Statistics, Ecological modelling, Macroecology

## Abstract

Locust population outbreaks have been a longstanding problem for Australian agriculture. Since its inception in the mid-1970s, The Australian Plague Locust Commission (APLC) is responsible for monitoring, forecasting and controlling populations of several locust pest species across inland eastern Australia (ca. two million km^2^). Ground surveys are typically targeted according to prevailing environmental conditions. However, due to the sheer size of the region and limited resources, such surveys remain sparse. Here we develop daily time-step statistical models of populations of *Chortoicetes terminifera* (Australian plague locust) that can used to predict abundances when observations are lacking, plus uncertainties. We firstly identified key environmental covariates of locust abundance, then examined their relationship with *C. terminifera* populations by interpreting the responses of Generalized Additive Models (GAM). We also illustrate how estimates of *C. terminifera* abundance plus uncertainties can be visualized across the region. Our results support earlier studies, specifically, populations peak in grasslands with high productivity, and decline rapidly under very hot and dry conditions. We also identified new relationships, specifically, a strong positive effect of vapour pressure and sunlight, and a negative effect of soil sand content on *C. terminifera* abundance. Our modelling tool may assist future APLC management and surveillance effort.

## Introduction

Locusts have been a longstanding challenge for many agricultural systems around the world^1^. In the temperate grain growing areas and semi-arid rangelands of eastern Australia, west of the great Dividing Range, *Chortoicetes terminifera*^2^ (Orthoptera: Acrididae) outbreaks frequently occur following enhanced grass productivity driven by above-average rainfall events^3–5^. These events often coincide with increased sea surface temperatures in the west Pacific Ocean (associated with La Niña) and/or the east Indian Ocean (negative Indian Ocean Dipole). There are several species of locusts in Australia that are of concern for Agriculture. In this paper, we focus on the most significant: *C. terminifera*, also known as the *Australian plague locust*. This endemic species is widespread throughout the continent, with frequent migration events evidenced by migration patterns and genetic studies^4,6^.

Locusts significantly affect yield of wheat, barley, oats and sorghum in Australia, with an estimated potential annual yield loss of 40% without intervention^7^. This translated into an economic agricultural loss of AUD$28.4 million in 2011 (US$19.8 million as of June 2020). Horticultural and pastoral industries are also subject to economic damage from locusts. The ability to monitor and forecast locust outbreaks is critical for managing their impact^8^. The main risks that locusts now present for Australia relate to control strategies. These need to account for the impact of control measures on ecosystems, people, animals and crops, albeit failure to control could still lead to economic and social impacts^9^. These concerns underpinned the establishment of the Australian Plague Locust Commission (APLC), which undertakes a programme of monitoring surveys throughout the geographical range of locusts in inland eastern Australia.

The population dynamics of *C. terminifera* are irruptive and thought to be driven by pulses in grass biomass productivity, driven largely by rainfall. Rainfall is highly variable in inland Australia but periods of rapid population increase generally follow heavy regional rainfall, provided local “seed” populations of locusts exist^3^. Every year, there tends to be three generations of *C. terminifera* north of 32°S and two in the south. Gregarious infestations can develop from sparse initial populations in a single year, following several generations of high recruitment^10^. Persistence during winter is assisted by facultative egg diapause induced in autumn, while summer quiescence in dry soils is an adaptation to drought^11,12^. A feature of locust outbreaks is frequent nocturnal, high altitude, wind-assisted migrations, with overnight displacements of several hundreds of kilometres^13,14^.

So far, modelling of *C. terminifera* has included two species distribution models^15,16^ and a process-based population dynamics model built using DYMEX (www.Hearne.Software)^17^. The species distribution models are binary models that aim to estimate whether a species is present or absent at a location, or whether it is an outbreak or not. These were applied to the case of predicting outbreaks under future climate change scenario using machine learning methods (random forest and boosted regression trees)^15^, and understanding the environmental and demographic dynamics of the specie with a state-space dynamic model^16^. Similarly, models of other species of locust have focused on predicting a categorical variable related to locust^18–20^. We note that one study examining the climatic drivers of locust plagues used a broadly similar to ours, albeit using long-term means rather than daily data^21^. Extending these models to estimates of abundance or density could enable a range of new ecological applications^22^. While promising algorithms exist to model abundance without consistent density surveys^23^, they are not needed for our study as we leverage survey data containing information on the density of nymphs and adults, albeit with some transformation (see Supplementary Material 1). This modelling approach allows us to model locust abundance directly, irrespective of their life-stage.

When studying pest species such as *C. terminifera*, there is often a disconnect between biological and ecological studies in a laboratory environment and large-scale population patterns. One of our aims is to assess whether such biophysical findings of locust abundance can be represented through a statistical model based on large-scale monitoring surveys. Thus, while our model does not include any explicit biological dynamics (such as gregariousness, or previous population), the relationships it finds between locust abundance and environmental characteristics can be used to infer the large-scale links between locusts and their environments. Previous studies have used climatic indices and data gridding^15,16^, although we note our covariates are gridded, in this study we leverage the survey data’s details by building a model specific to each survey site’s location, up to a daily resolution.

Locust development is a complex phenomenon, which involves many non-linear processes^24^, where different environmental conditions could lead to similar abundances. In this paper we model the abundance of *C. terminifera* using both Generalized Linear Models (GLM) and Generalized Additive Models (GAM). We also use feature selection algorithms with GLMs to narrow down a set of 10 covariates to investigate further. As GAMs incorporate non-linear data, these models have gained popularity in the ecological modelling community^25,26^. These models have been shown to capably model ecological processes while remaining easy to interpret^27,28^.

Our study aims to: (a) quantify the relative strength of various biophysical variables driving locust population fluctuations (b) develop data-driven models of locust abundance based on these variables and (c) illustrate optimal model performance and uncertainties through bivariate maps. The end-goal is to build towards a forecasting system for locust populations, to enable practitioners to see evolving hazards and direct their survey efforts, and to support their locust management strategy.

## Methods

### Datasets

#### Survey data

This study is based on a database of field surveys conducted by the Australian Plague Locust Commission (APLC) over the last three decades. Although surveys occurred earlier, we limit our analysis to years from 1992 to 2019, as these coincide with the availability of satellite vegetation maps and the introduction of GPS for survey locations (significant improvement in localisation). These surveys monitor the locust situation across the Australian states of Queensland, South Australia, Victoria, and New South Wales. As such they aim to inform control efforts and avoid outbreaks. Although not the focus of the collection, these surveys provide valuable long-term data on locust populations. These datasets can help inform our understanding of locust populations, and their environmental drivers. The APLC survey data have been used in previous studies^15,16^, which focused on seasonal outbreak predictions.

The APLC uses a rapid, roadside-based index monitoring surveillance system to assess locust populations and to detect areas of high densities with the potential to affect interstate agriculture that could warrant control intervention. Surveys are conducted during the austral spring–autumn period (September to May), with sampling units at ~ 10 km intervals. During winter the bulk of the locust population is undetectable, as eggs are dormant and few surveys are conducted. The accumulated data records are structured with a bias to favourable grassy habitats and ancillary report information, they are also subject to large temporal and spatial discontinuities. However, they provide the only spatio-temporal record of population changes in sampled areas and are spatially accurate to within 0.01° of latitude and longitude.

**Figure 1:**
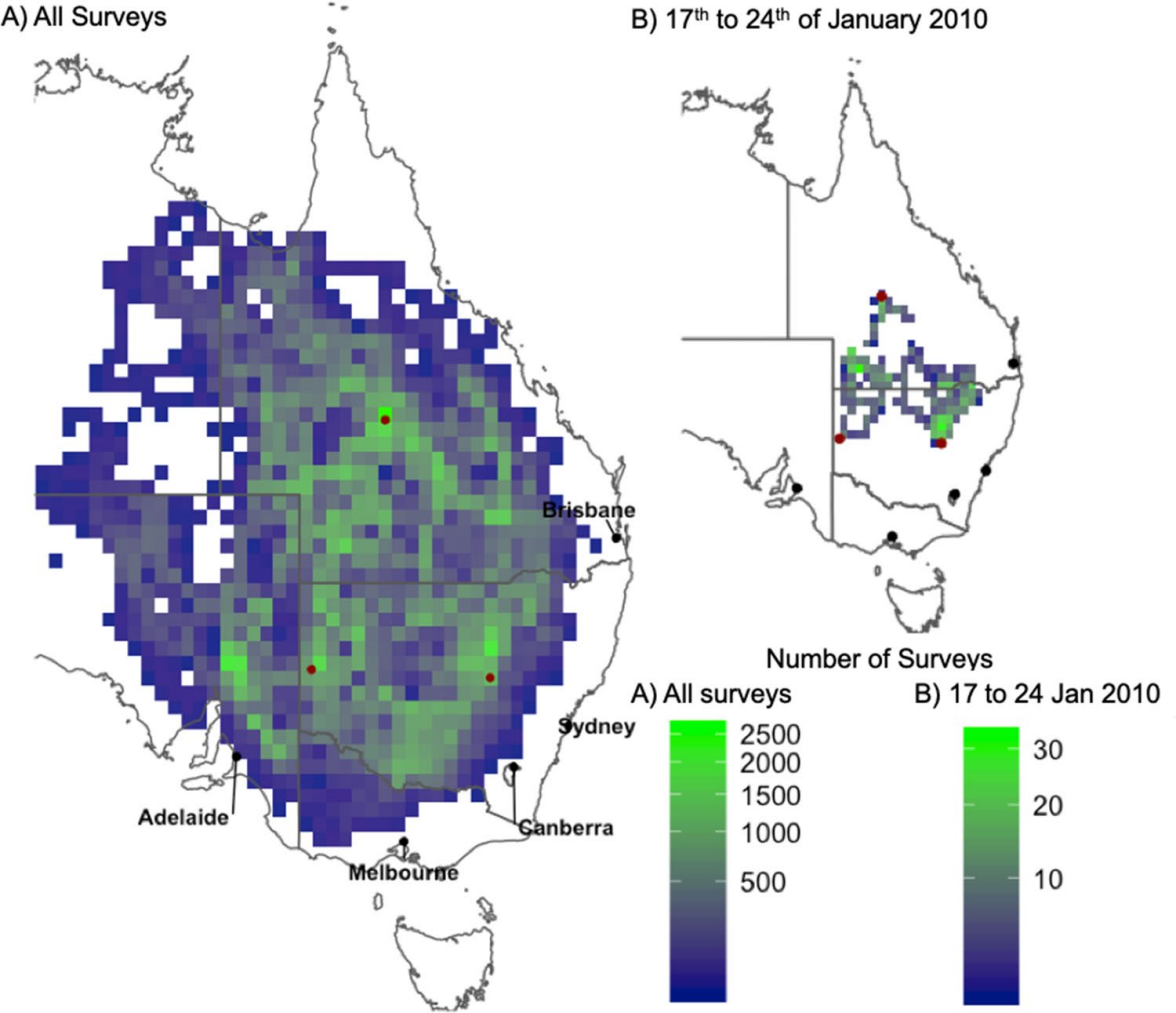
The APLC survey spatial coverage for all surveys from 1992 to 2019 (**A**, left), and its (illustrative) reduced coverage for 1 week in January 2010 (**B**, top right) gridded to 0.5 × 0.5 degrees bins showing the number of surveys. APLC field bases are shown as red dots, these are (clockwise from the top): Longreach, Narromine, and Broken Hill. We expect our model to perform better in areas that were heavily surveyed. Maps produced in R (v3.6.0) with the ggplot, ozmaps and sf libraries (see https://cran.r-project.org/).

The dataset contains about 295,238 observations and their spatial coverage can be seen in Fig. 1. We find that each year, on average 32% (σ = 11%) of the surveys observed locusts. This may be an overrepresentation of locust presence in Australia, as the surveys are biased towards habitats favourable to locusts. Furthermore, the surveys have logistical bias: they are concentrated near roads, and more frequently around APLC field bases (e.g., Longreach in Queensland) as visible in Fig. 1. While the dataset covers most of eastern Australia our analysis period (1992 to 2019), coverage is limited spatially over shorter timespans, as illustrated by one week in January 2010 (Fig. 1B). This lack of spatial coverage highlights the usefulness of a model to “fill in the gaps” in survey data. The categorical data of the surveys are transformed into continuous abundance data by sampling from a normal distribution (for further details, see Supplementary Material 1), this introduces a ceiling on our performance with a standard deviation of at best 30% of the mean estimate.

#### Ancillaries and covariates

Weather variables were obtained from the SILO archive of Australian climate data, interpolated onto a 0.05 arc degree grid^29^. In this study we use the data as-is, though we note such interpolations are uncertain, particularly in regions where observation stations are sparse^30^. We note the original weather interpolation used a tri-variate thin plate smoothing spline, the independent variables they used were latitude, longitude and elevation. See the original paper^29^ for the implications of this interpolation on the data and an assessment of the related correlation structure. For each entry in the survey database, we extracted: maximum daily temperature (°C), daily rain (mm), minimum temperature (°C), vapour pressure (hPa, in meteorological data this refers to the partial pressure of water vapour in the atmosphere), and solar radiation (total incoming shortwave, MJ m^−2^). We then take both short-term (10 days) and long-term (60 days) prior values of these variables, and their summary statistics: minimum (min), maximum (max), and mean. For instance, if a survey occurred on the 15th of March 2010, the short-term memory mean daily rainfall would be the average daily rainfall from the 5th of March to the 15th of March 2010, while the long-term memory would be the average daily rainfall from the 14th of January to the 15th of March 2010. If a survey was done at the same location 1 day later, these memory values would shift forward by 1 day. 10 and 60 days were guesses, somewhat informed by the lifespans of *C. terminifera*^2^*.* Besides these summary statistics, we also create additional covariates that reflect the number of days that experienced no rainfall (the number of dry days) or extremes in temperature: a daily minimum below 18 °C and maximum above 42 °C. We adapted these temperature thresholds from those used in^31^. This provides our algorithm with covariates similar to degree-days or environmental thresholds detrimental to insect development, these are commonly used in estimating pest abundance^32^. Note we ignored the minimum daily rain, as respectively all of the long-term and the vast majority of the short-term were 0 mm day^−1^. In addition to these weather variables, we include monthly Normalised Difference Vegetation Index (NDVI) for Australia from 1992 onwards. This product is obtained from the Advanced Very High Resolution Radiometer (AVHRR), and aggregated onto a 0.05° grid^33^. We removed any negative NDVI from our analysis (0.2% of our data). We use static maps (30 m resolution) for soil properties (fraction of clay, sand, and silt) from the Soil and Landscape Grid of Australia^34^. Finally, our GAMs take into account survey location in time and space (day of the year, longitude, and latitude), in order to account for high spatial heterogeneity and local effects not accounted for by our covariates.

To accommodate the various native resolutions of our covariates, we take the covariate value at the location nearest each survey site. Thus, each surveyed density is deemed an independent sample with an associated set of covariates, unique to its date and location. For inference purposes (our “Results” section) some of our variables were highly correlated (see Table S2) and many of our covariates relate to similar physical quantities, for instance, we have 16 covariates related to temperature alone (see Table 1). We tried to account for these biases through our selection of a reduced set of covariates for our models.

**Table 1:**
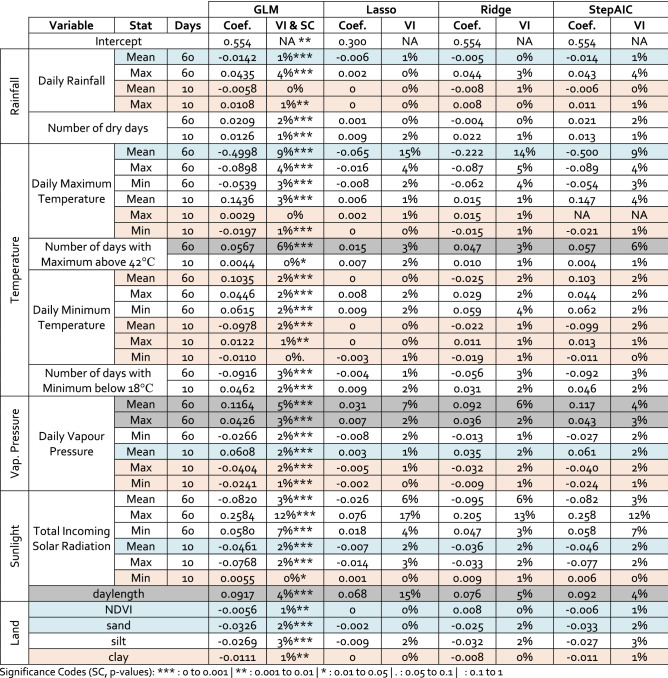
Significant variables for *Chortoicetes terminifera* density.

Significance Codes (SC, p-values): *** : 0 to 0.001, ** : 0.001 to 0.01, * : 0.01 to 0.05, . 0.05 to 0.1, : 0.1 to 1.

**Figure 2:**
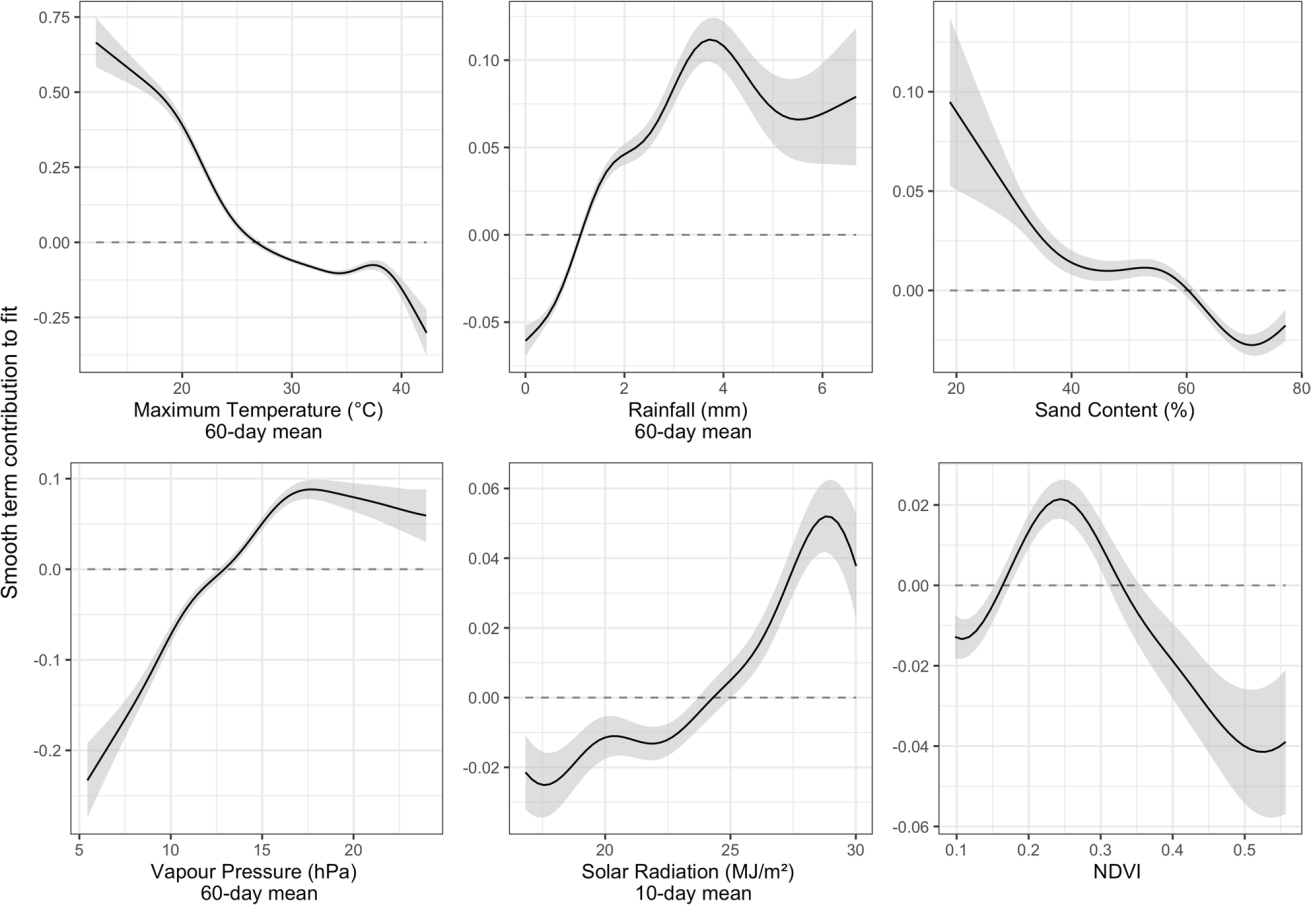
The response curves of our “all densities” GAM (GAM#2 in Table 2) for a reduced set of 6 variables of ecological importance. Note the varying y-scales. Our biophysical values (x-axis) were bound by a signal to noise ratio of 2 (standard error less than half the response curve). The solid line shows the model fit, and the shaded area shows 2 standard deviations. Notwithstanding the influence of other covariates, these plots can be used to interpret the apparent influence of each covariate on the observed abundance of *Chortoicetes terminifera*. A positive smooth contribution corresponds to higher estimated abundance, a negative one to lower abundance. Large shaded areas can be due to an uncertain relationship, or a lack of data. A null response (y = 0) is shown with a dashed line to facilitate interpretation. This figure can also be viewed as what the function that relates each covariate (independently) to the density of locust would look like.

Variable Importance (VI) is based on the t-statistic of each model (normalized to 100%). The subset of 6 variables of interest we use in Fig. 2 for the purpose of our GAM’s interpretation is shaded blue. We exclude the 12 variables shaded red from our model selection and comparison (Table 2). Finally, the set of 4 variables shaded grey were excluded from Fig. 2, but included in the GAM#2 models (see Supplementary Material Figure S1 and S2 for the full set of variables).

### Statistical techniques

In “Datasets” section, we show some statistical analysis of the APLC survey dataset for context. In “Statistical techniques” section, the focus of our work, we model the density of *C. terminifera* using environmental covariates. We firstly pre-process the data, then for GLMs we scale the ancillaries so that each variable has a mean of 0 and standard deviation of 1. This scaling is not necessary for the GAMs. In addition, to reduce the skewness of their distributions, we also use a power transform or sixth root transform for our estimatid value, such that our models are estimating (densities)^1/6^. Typically, densities would be log-transformed, however, we chose to use a power transformation (with *λ* = 1/6 = 0.167) to account for observations of 0 densities (absence of locust). These pre-processing steps allow us to have data distributions more appropriate for statistical algorithms when fitting our models^35,36^.

We use a training/validation split of 80:20%, and tenfold cross-validation for our GLM (Table 1). The training set corresponds to a (randomly) sampled fraction of all the data on which our models are trained or fitted. Their performance is then assessed on a separate validation set that the models had not been trained on. Cross-validation algorithms similarly divide the data, although it was restricted to the model training step, and a means to avoid overfitting. The data for the training and test set are randomly sampled from all the available survey locations and time. This effectively means that our models are trained on 72% of the data, tested on a different 8% ten times over until a best model was found. Our final model performance is assessed on the remaining 20%, which was completely unknown to the model though still correlated with the training dataset.

We performed feature selection using four Generalized Linear Model (GLM)^37^ algorithms. These include standard GLM, lasso and ridge regression^38^ and stepwise regression using the Akaike Information Criterion (StepAIC)^39^. The Variable Importance (VI) was derived using Student’s *t*-statistic, normalized so that all VIs summed to 100%. Once we had narrowed down a subset of 10 useful covariates, we built GLMs and GAMs, which we compared using three metrics: the Akaike Information Criterion (AIC), R-squared (R^2^) and the Root Mean Square Error (RMSE). We also use this subset to interpret the relationships between abundance and covariates (Fig. 1, as well as Supplementary Material Figures S1, S2, and S3).

Generalized Additive Models (GAMs) were built using a specialised numerical implementation for large datasets^40^. The use of GAMs allowed us to include non-linear interactions between the covariates and the estimates (*C. terminifera* densities). These models were configured such that the response functions were taken from the negative binomial family, with a square-root link function. We use a space–time tensor (a 3D matrix to represent the effect of location and time-of-year) that varies with (unscaled) longitude, latitude, and day of the year. In essence, this tensor represents the (interacting) contribution of location in space and time to our models, and also highlights spatial and temporal correlation patterns in the data we used. For further details and mathematical formulae, we point the reader to our Supplementary Material and Eq. 1 in particular. All other variables were implemented using spline smooths, with no interacting covariates. For instance, the temperature has no effect on the model fitting of the vapour pressure, and vice versa.

## Results

### Statistical models

Based on four GLMs commonly used for feature selection, we explored the most significant model variables (Table 1). From these we selected 10 covariates: prior rainfall and maximum temperature (60-day mean), the number of days above 42 °C, prior vapour pressure (60-day mean and max, 10-day mean), the incoming solar radiation (10-day mean), the current daylength, NDVI value (index between 0 and 1) and soil sand content (as a % of soil content; Table 1). These 10 covariates were either important to all methods as evidenced by their Variable Importance in Table 1, or deemed of particular ecological or logistical interest (NDVI and soil sand content). In contrast, we removed 12 variables based on their low t-statistic across our four models, their high correlations with other variables (see Supplementary Material Table S2) or on their lack of ecological interest. This left our “full” models (#1 in Table 2) with 27 variables (so that with the 3D tensor, we had a total of 30 variables—note this choice was heuristic, and not based on hyperparameter optimization).

**Table 2:** Performance metrics for the various statistical models used to estimate density of *Chortoicetes terminifera*.

Model	Num. variables	AIC	R^2^	RMSE
**Nymph density (no zeroes, or 8.25% of surveys)**				
GLM #1	27	28,323	0.311	0.392
GLM #2	10	29,718	0.275	0.402
GAM #1	27 (+ 3)	72,700	0.461	0.562
GAM #2	10 (+ 3)	72,697	0.451	0.563
**Adult density (no zeroes, or 57.67% of surveys)**				
GLM #1	27	48,606	0.075	0.299
GLM #2	10	51,502	0.054	0.303
GAM #1	27 (+ 3)	212,113	0.175	0.334
GAM #2	10 (+ 3)	212,438	0.144	0.337
**All densities (zeroes & nymph & Adult)**				
GLM #1	27	374,015	0.144	0.548
GLM #2	10	365,083	0.107	0.559
**GAM #1**	**27 (+ 3)**	**369,318**	**0.286**	**0.544**
*GAM #2*	*10 (+ 3)*	*371,027*	*0.271*	*0.549*

In the number of variables column, (+ 3) relates to the inclusion of space (latitude and longitude) and time (day of the year). The bold values GAM (#1, all densities) represents the model used in Fig. 4 for spatial estimations, while the italic values GAM (#2) is shown in Fig. 2 for interpretation purposes.

Table 2 shows the performance metrics of our models, for models with either a reduced set of covariates (#2, the 10 grey and blue covariates in Table1) or a full set (#1, the 27 not red covariates in Table 1). The GAM with spatio-temporal tensor and all covariates (GAM #1) performed best explaining 28.6% of the deviance, while the Nymphs-only GAM #1 model explained 46.1% of the deviance. Importantly, the nymph model was fitted using only the subset of surveys where nymphs were observed (8.25% of all data). The R^2^ values in Table 2 are somewhat small, which could be due to a noisy dataset and complexities that our models cannot account for. We also introduced a random sampling to convert density classes into real densities (see supplementary material), which caps our estimation power. Nonetheless, low R^2^ values are common with abundance estimates^28^. Performing ranked correlation for our core model (GAM #1), we found a Spearman coefficient ρ = 0.331 and a Kendall coefficient τ = 0.237. The scatterplot in Supplementary Material Figure S5 illustrates these by highlighting the model’s difficulty in accurately estimating high densities. This suggests our approach is unlikely to accurately estimate outbreaks in the correct magnitudes.

### Model interpretation and discussion

These models can be leveraged to interpret the statistical relationships between each of our covariates and locust densities, allowing us to infer the influence of the covariate on the ecological niche of *C. terminifera*. In the case of linear relationships between covariates and locust density, this is readily visible through the GLMs presented in Table 1, where the coefficients columns show whether the covariate encourages (positive coefficients), or discourages (negative coefficients) high densities of *C. terminifera*. Hence, we observe some broad relationships: populations are higher when daylengths exceed 12-h (spring and autumn) and vapour pressure is high (reflecting humidity). In contrast, the long-term mean maximum temperature is likely to have a negative impact on locust densities. This may reflect seasonality of rainfall, as well as heat-related mortality.

However, there are important caveats to interpreting linear coefficients in isolation. Firstly, our statistical method compounds the influence of each covariate, which is problematic when using many variables. Secondly, these models are best at identifying linear relationships between covariates and estimates. In contrast, GAMs account for non-linear relationships better. To address both of these issues, our model interpretation will focus on the response curves of a GAM with a reduced set of the 10 most influential covariates (GAM #2 in Table 2), 6 of which we show in Fig. 2 and use in our interpretation.

Figure 2 illustrates six covariates that are of particular interest, in zones of low uncertainty, while Supplementary Material Figure S1 and S2 use all 10 covariates and include zones of large uncertainties, and Figure S1 focuses on Nymphs. Lastly, Supplementary Material Figure S3 shows the spatio-temporal tensor of both of our GAMs (for nymphs only and all *C. terminifera* lifestages). In the smooth term contribution to fit of Figs. 1, S1 and S2, a positive response (y-axis above 0) indicates a corresponding increase in the density estimate, while a negative response would decrease it. In the case of Supplementary Material Figure S3, this is shown through the colours (or z-axis), going from red for a negative response, to yellow for a positive one. Additional information to aid in interpreting and understanding our GAMs and these Figures can be found in the Supplementary Material. Note that while daylength is identified as being very important in Table 1, it does not contribute as strongly in the GAM. This is because daylength varies with latitude and time of the year, which are already accounted for by our spatio-temporal tensor. Figure 2 also includes the uncertainty in the relationships between covariates and estimate with shaded areas (representing 2 standard deviations). Model uncertainty could be related to uncertain relationships and/or lack of data. To aid interpretation in Fig. 2, we restrict our figures to regions where the standard error is less than the estimated response. To further simplify the interpretation, particularly for the figures in the Supplementary Material (Figure S1 and S2), we suggest focusing on areas with low uncertainty (narrow shaded area), and to assess whether the covariate encourages (positive values) or discourages (negative values) abundance of *C. terminifera*.

The relationship between the 10 covariates identified and *C. terminifera* abundance reflects the current understanding of habitat productivity and ecophysiological limitations in the biology of locusts in Australia: higher rainfall over the long-term and high vapour pressure (through humidity) increases the amount of food resources available for locusts^41^. High vapour pressure generally means higher relative humidity, although this relationship is confounded by temperature. Interestingly, both long-term daily rainfall (60-day mean) and vapour pressure (60-day max) show a peak of around 4 mm day^−1^ and 18 hPa respectively. This may reflect that some rainfall and humidity is beneficial to *C. terminifera* population growth, up to a threshold where conditions would become less hospitable. This pattern is consistent with the Law of Tolerance^42^, which states that organisms have environmental or biophysical thresholds (minimum, maximum and optimum) which determine their success. Meanwhile, Xeric conditions (low rainfall and vapour pressure) are associated with lower density locust populations, which may be related to reduced food availability.

Supplementary Material Figure S1 and S2 show divergent relationships with NDVI between adults and nymphs, with larger adult populations at very low and high NDVI, where suitability peaks around 0.25 before decreasing up to 0.6 and then increasing again. This bimodal response suggests that there is a confounding effect in the response of *C. terminifera* abundance to NDVI. This may be the result of high NDVI vegetation types, such as forest, being largely avoided for oviposition. Such confounding interference could perhaps also come from spatial or temporal mismatch, for example, due to migration: the abundance of a cohort of locusts that has migrated into a region was not dependent on that region’s observed characteristics, but on that of the region from which they originated. Seasonal trends in NDVI have already been used to monitor locust habitat^43^. Our analysis also shows that sandy soils and areas with high NDVI (a proxy for vegetation cover) rarely support a high density of nymphs, which we interpret as these areas being unlikely oviposition sites.

Daily maximum temperature (60-day mean) is associated with decreased abundance of *C. terminifera* populations. Woodman^24^ studied the negative effect of very high temperatures (> 45 °C) on first instar nymph survival rate, but also posited that *C. terminifera* are robust to high temperatures alone. This is reflected in the positive impact of number of days above 42 °C on *C. terminifera* abundance. Note these relationships between temperature and *C. terminifera* abundance may also reflect seasonality. Finally, sunlight (10-day mean solar radiation) appears to promote the abundance of *C. terminifera*, likely by enhancing plant productivity or increasing the period locusts can optimise body temperature by basking.

**Figure 3:**
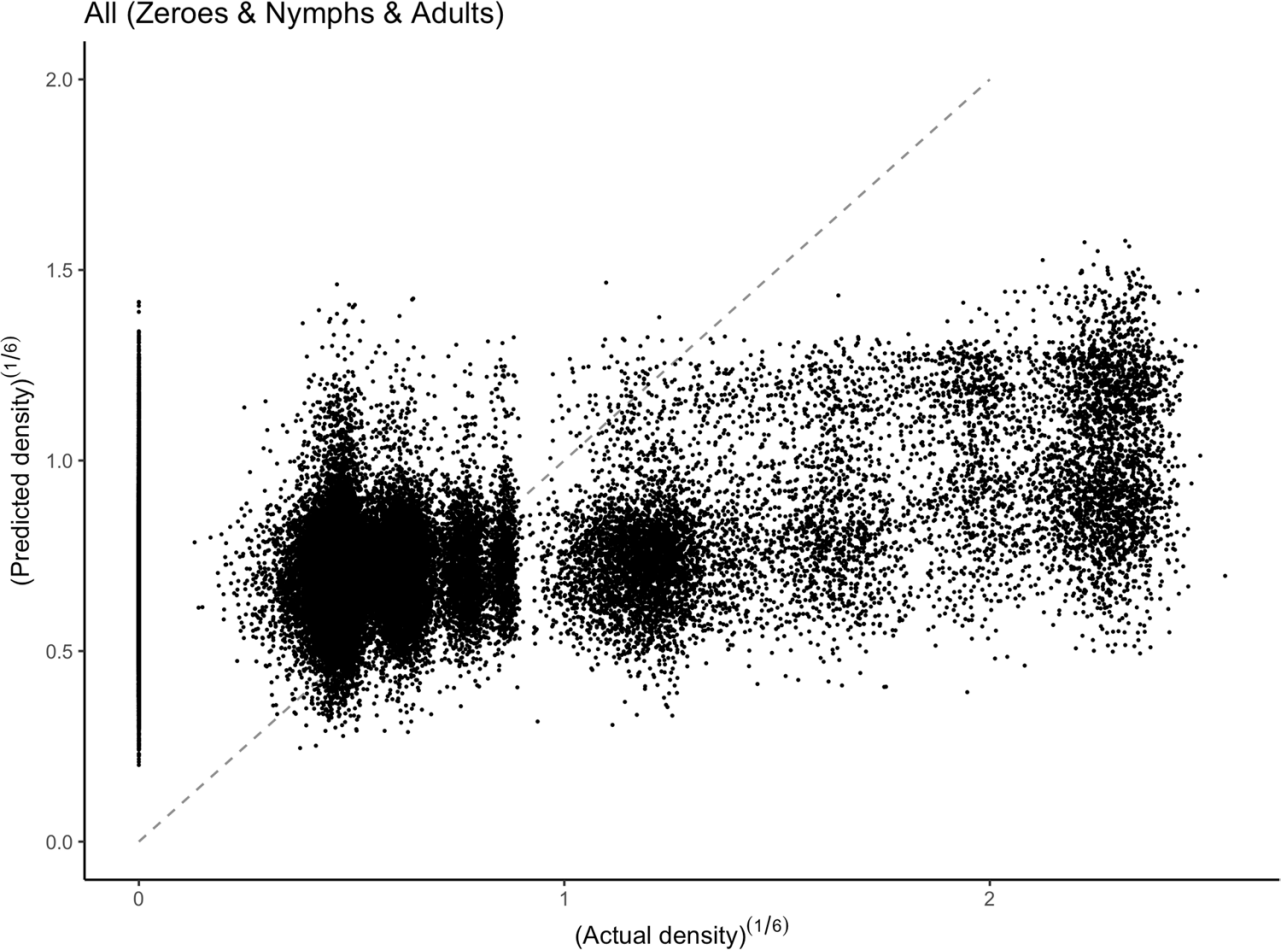
A scatterplot showing observed vs predicted densities for our model of all locust lifestages. The dashed line shows the identity line (observed equals estimated). We can see the model tends to overestimate densities below 0.6, and zero densities in particular. In contrast, the model strongly underestimates the densities above 1.

Lastly, the biases in the magnitude of our predictions can be assessed in Fig. 3 and Supplementary Material Figure S6. Of particular relevance when predicting outbreaks, the model performs best when estimating densities around 0.6 individuals per m^2^, and is less accurate for low (zero in particular) and high densities. In part, this model bias can be explained by the fewer surveys of high densities (see Supplementary Material Table S1). Due to the use of a 6th root transform, these effects drastically impact the estimate of high-densities. We also note our models are not able to replicate zero densities, which is consistent with the type of model used. Future approaches that leverage zero-inflated distributions might be able to address this. Note the “Nymphs Only” models do not have this zero-inflation (this is a result of survey design, not population dynamics). This also explains the higher performance of the Nymphs model. While our models were able to reproduce the general increase of population numbers with environmental conditions, they underestimated the sharpness of this increase.

### Mapping our estimates

**Figure 4:**
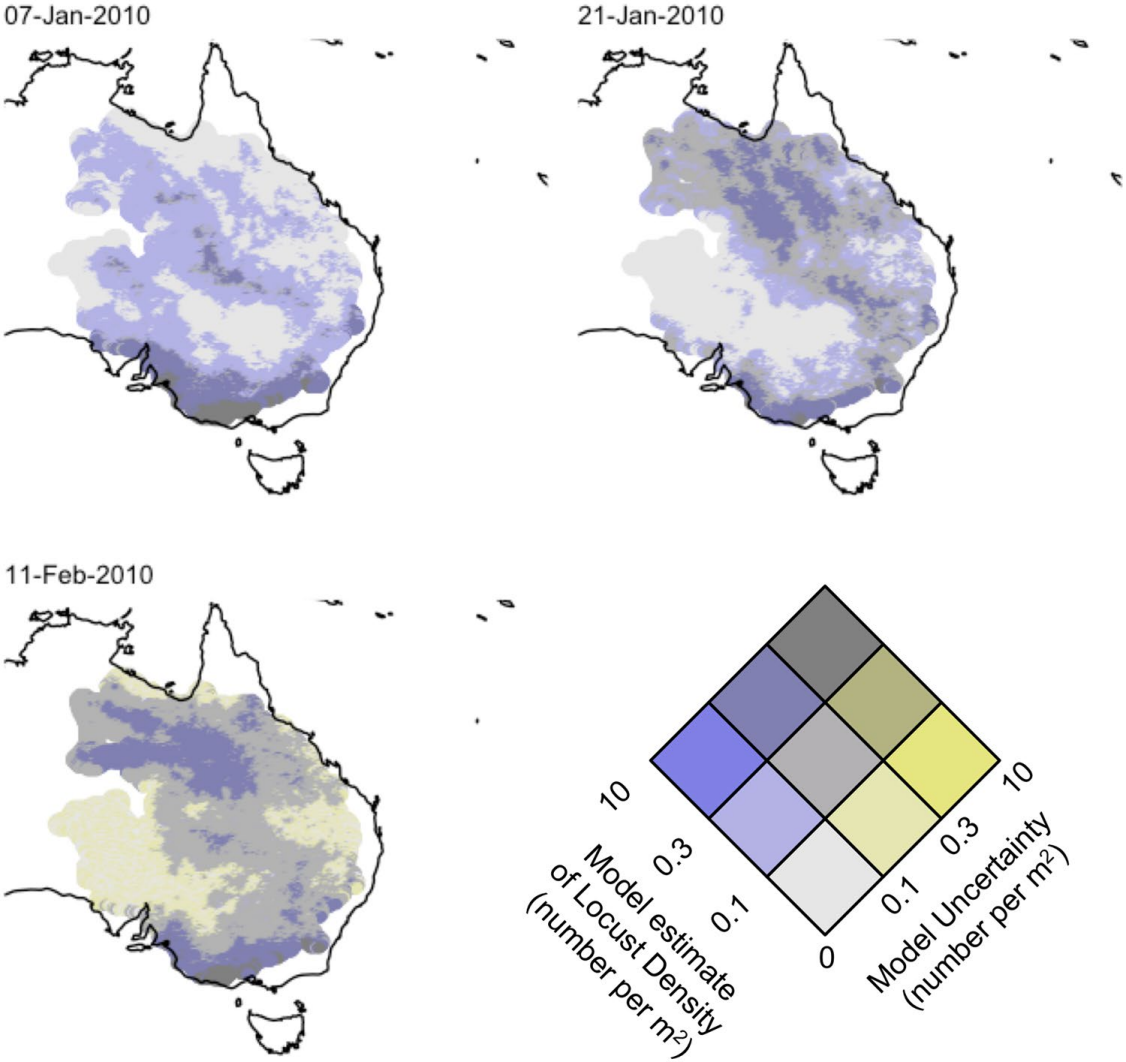
Model estimates of abundance of *Chortoicetes terminifera* throughout eastern Australia for three days in 2010 shown through bivariate maps of our GAM (GAM #1 with Adults and Nymphs, blue shade in Table 2) displaying both the density of locust and associated uncertainty (as a fraction of the estimated abundance). Our estimates are restricted to locations within 50 km of sites that were surveyed at some time. Bright blue areas correspond to estimates of high density with low uncertainty, bright yellow to high uncertainty and low density, grey (blue + yellow) to high density with high uncertainty. Maps produced in R (v3.6.0) with the ggplot and VizuMap libraries (see https://cran.r-project.org/ and ^44^).

The statistical models we developed allow us to use environmental covariates to estimate abundance of locusts at any location within eastern Australia. Figure 4 shows the estimated density of locusts throughout eastern Australia for three days in 2010 during an outbreak season. Locations that were never visited by Australian Plague Locust Commission (within 50 kms) were excluded. The figure was produced leveraging the Vizumap package for R^44,45^. By merging the modelled estimates of *C. terminifera* abundance and the uncertainty therein, we can interpret model results and confidence levels. The uncertainty component includes both the standard error from the model, as well as that due to the random sampling to convert survey density classes into abundance of *C. terminifera*. See Supplementary Material for more details.

Model estimates of spatial patterns differ across January–February in 2010. In Fig. 4, we illustrate this by showing dates a few weeks apart, with distinctive patterns in estimated densities. Overall, the model’s highest (and most uncertain) *C. terminifera* density estimates tend to be located in south-eastern Australia. As time progresses from the 7th of January to the 11th of February, our model estimates an increasing locust population in the North, with higher numbers on the 11th of February, albeit with higher uncertainty. This is reflected through yellow tones, leading to more brown for high density estimates and yellows for low-density yet uncertain estimates.

Our method is somewhat sensitive to the APLC field survey protocols, notably through: (1) the random uncertainty introduced by sampling from abundance categories, and (2) the spatio-temporal coverage of the surveys. Surveys were focused on areas considered likely to host *C. terminifera* and as a consequence our statistical models may overestimate their range of habitats. The high estimates in south-eastern and northern Australia may be an artefact of this survey bias, which can be seen in Fig. 2 as areas surveyed less often or not at all. To allow readers to explore the differences between the survey and our model’s estimates, we included additional figures in the Supplementary Material. The geographical differences can be explored in the map of Root-Mean-Squared-Error in Supplementary Material Figure S5.

## Conclusions

Quantitative predictive models of Locust abundance could lead to improved management decisions and positive impacts on Australian agriculture. In this study we show how statistical models can help estimate Locust abundance on a large scale that is relevant to coordinated broadscale area-wide pest management. Furthermore, we modelled abundance as a continuous variable (locust density) rather than a density category (absence–presence-swarm). Continuous variables such as this are more representative of the population dynamics at play within the landscape. By tracking covariates at each survey site in daily timesteps, we build models that represent the biophysical influences onto locusts with as fine a detail as possible.

Our models show that environmental covariates alone can be used to forecast the abundance of locust with a reasonable amount of skill. We found better performance when modelling nymphs (R^2^ = 0.461) rather than all lifestages of locusts (R^2^ = 0.286). This may reflect the stronger dependence of nymphs on their local environment, as well as the dispersion capabilities of adult locusts. Locust abundance appears to follow non-linear relationships with our covariates, which is illustrated by the better performance of GAM rather than GLM (R^2^ = 0.144 for all lifestages, R^2^ = 0.311 for nymphs). The response curves shown in Fig. 2 (as well as S1 and S2) highlight how each independent variable influences estimates of locust densities. This may include windows of environmental conditions favourable to locust population growth. For example NDVI around 0.25 favours higher locust abundance, echoing previous findings^43,46^. We then leverage that model to estimate locust abundance across eastern Australia during the 2010 season. Figure 4 illustrates how our statistical models can be used to not only estimate areas of outbreaks, but also to assess the associated uncertainty in these estimates.

Our models may be improved by including more covariates, or more likely the interaction between covariates, for instance temperature and vapour pressure. While our study looked at the surveys from all of Australia, our approach could be used for regional subsets, for more accurate local estimates in regions of particular interest. Another improvement could be the inclusion of ecological processes such as locust migration, or the abundance levels of previous generations. This could account for the compounding nature of population growth, and improve the estimates of low and high abundance levels. The higher performance of our model at estimating the density of nymphs suggests that environmental variables, at least the ones we include, are more important to the development of *C. terminifera* nymphs than adults. This might be related to the dispersal ability and more ubiquitous distribution of adult locusts, and the specificity of local habitat conditions for egg and nymph survival. We note that state-space models^16^ could be suited for modelling these processes explicitly. The higher performance of the nymph-only model may also reflect the greater sensitivity of nymph populations to their environments than adults^14^.

We recommend three avenues to improve the estimates of locust abundance in eastern Australia. Firstly, a Machine Learning approach could yield better performance at modelling locust abundance or outbreaks^15^. Care should be taken when interpreting such models however, as Machine Learning can be unsuitable for inference^47^. Secondly, ongoing and historical surveys could be included when estimating abundance, either as statistical covariates or through a data assimilation framework. Note this would be less useful for forecasts or for estimates in areas where surveys are rare. Thirdly, more abiotic covariates may be included to improve model fit. This could involve high-resolution soil moisture data from satellites, as soil moisture impacts egg laying, egg quiescence and nymph survival through food availability^48^. While our models did not identify NDVI as a key variable, other measures of vegetation productivity such as Fraction of Absorbed Photosynthetically Active Radiation (FAPAR)^49^ or tree cover data may be important covariates, as vegetation type may reflect different foods available to locusts at each life-cycle stage. Lastly, future models could also include atmospheric patterns to reflect the long-range dispersal of adult locusts by wind^50^.

Here we demonstrate how a statistical framework may be used to leverage extensive survey efforts into estimates of abundance. We show how non-linear statistical models forced by a suite of biophysical variables can lead to more accurate estimates of locust abundances than equivalent linear models, highlighting their potential to assist pest management decision-making. We explore the use of bivariate maps to display not only abundance estimates, but also confidence levels. Including uncertainties in our models can aid judgement in their use for operational purposes, and in their inclusions into forecasts for pest management.

## Supplementary information

Supplementary File 1.

## References

[1] Stige LC, Chan K-S, Zhang Z, Frank D, Stenseth NC (2007). Thousand-year-long Chinese time series reveals climatic forcing of decadal locust dynamics. Proc. Natl. Acad. Sci..

[2] Walker, F. Catalogue of the Specimens of *Dermaptera Saltatoria* in *Collection of the British Museum. Part III.* 485–594 (British Museum (Natural History), 1870).

[3] Wright DE (1987). Analysis of the development of major plagues of the Australian plague locust *Chortoicetes terminifera* (Walker) using a simulation model. Aust. J. Ecol..

[4] Deveson ED, Walker PW (2005). Not a one-way trip: Historical distribution data for Australian plague locusts support frequent seasonal exchange migrations. J. Orthoptera Res..

[5] Wang, H. Quantitative assessment of Australian plague locust habitats in the inland of eastern Australia using RS and GIS technologies in *Remote Sensing for Agriculture, Ecosystems, and Hydrology XVI* vol. 9239 92390D (International Society for Optics and Photonics, 2014).

[6] Chapuis M-P (2011). Challenges to assessing connectivity between massive populations of the Australian plague locust. Proc. R. Soc. B Biol. Sci..

[7] Murray DAH, Clarke MB, Ronning DA (2013). Estimating invertebrate pest losses in six major Australian grain crops. Aust. J. Entomol..

[8] Zhang L, Lecoq M, Latchininsky A, Hunter D (2019). Locust and grasshopper management. Annu. Rev. Entomol..

[9] Adriaansen C, Woodman J, Deveson E, Drake V (2016). The Australian Plague Locust: risk and response. Environ. Hazards Risks Disasters Biol.

[10] Farrow RA, Longstaff BC (1986). Comparison of the annual rates of increase of locusts in relation to the incidence of plagues. Oikos.

[11] Wardhaugh KG (1980). The effects of temperature and moisture on the inception of diapause in eggs of the Australian plague locust, *Chortoicetes terminifera* Walker (Orthoptera: Acrididae). Aust. J. Ecol..

[12] Wardhaugh KG (1986). Diapause strategies in the Australian plague locust (*Chortoicetes terminifera* Walker). The evolution of insect life cycles.

[13] Clark DP (1971). Flights after sunset by the Australian plague locust, *Chortoicetes terminifera* (Walker) and their significance in dispersal and migration. Aust. J. Zool..

[14] Farrow RA (1977). Origin and decline of the 1973 plague locust outbreak in central western New South Wales. Aust. J. Zool..

[15] Wang B (2019). Future climate change likely to reduce the Australian plague locust (*Chortoicetes terminifera*) seasonal outbreaks. Sci. Total Environ..

[16] Veran S (2015). Modeling spatiotemporal dynamics of outbreaking species: influence of environment and migration in a locust. Ecology.

[17] Maywald, G., Kriticos, D., Sutherst, R. & Bottomley, W. *DYMEX model builder version 3: user’s guide*. (2007).

[18] Meynard CN (2017). Climate-driven geographic distribution of the desert locust during recession periods: Subspecies’ niche differentiation and relative risks under scenarios of climate change. Glob. Change Biol..

[19] Piou C (2013). Coupling historical prospection data and a remotely-sensed vegetation index for the preventative control of Desert locusts. Basic Appl. Ecol..

[20] Tratalos JA, Cheke RA, Healey RG, Stenseth NC (2010). Desert locust populations, rainfall and climate change: Insights from phenomenological models using gridded monthly data. Clim. Res..

[21] Tian H (2011). Reconstruction of a 1,910-y-long locust series reveals consistent associations with climate fluctuations in China. Proc. Natl. Acad. Sci..

[22] Ehrlén J, Morris WF (2015). Predicting changes in the distribution and abundance of species under environmental change. Ecol. Lett..

[23] Croft S, Chauvenet AL, Smith GC (2017). A systematic approach to estimate the distribution and total abundance of British mammals. PLoS ONE.

[24] Woodman JD (2011). High-temperature survival is limited by food availability in first-instar locust nymphs. Aust. J. Zool..

[25] Guisan A, Edwards TC, Hastie T (2002). Generalized linear and generalized additive models in studies of species distributions: Setting the scene. Ecol. Model..

[26] Yee TW, Mitchell ND (1991). Generalized additive models in plant ecology. J. Veg. Sci..

[27] Bučas M (2013). Empirical modelling of benthic species distribution, abundance, and diversity in the Baltic Sea: Evaluating the scope for predictive mapping using different modelling approaches. ICES J. Mar. Sci..

[28] Heersink DK (2016). Statistical modeling of a larval mosquito population distribution and abundance in residential Brisbane. J. Pest Sci..

[29] Jeffrey SJ, Carter JO, Moodie KB, Beswick AR (2001). Using spatial interpolation to construct a comprehensive archive of Australian climate data. Environ. Model. Softw..

[30] Tozer CR, Kiem AS, Verdon-Kidd DC (2012). On the uncertainties associated with using gridded rainfall data as a proxy for observed. Hydrol. Earth Syst. Sci..

[31] Gregg P (1983). Development of the Australian Plague Locust, Chortoicetes terminifera, in relation to weather I. Effects of constant temperature and humidity. Aust. J. Entomol..

[32] Pruess KP (1983). Day-degree methods for pest management. Environ. Entomol..

[33] McVicar TR, Briggs PR, King EA, Raupach MR (2003). A review of predictive modelling from a natural resource management perspective: the role of remote sensing of the terrestrial environment.

[34] Grundy MJ (2015). Soil and landscape grid of Australia. Soil Res..

[35] Cressie N, Wikle CK (2015). Statistics for spatio-temporal data.

[36] James G, Witten D, Hastie T, Tibshirani R (2013). An introduction to statistical learning.

[37] Nelder JA, Wedderburn RW (1972). Generalized linear models. J. R. Stat. Soc. Ser. Gen..

[38] Friedman J, Hastie T, Tibshirani R (2010). Regularization paths for generalized linear models via coordinate descent. J. Stat. Softw..

[39] Venables WN, Ripley BD (2002). Modern Applied Statistics with S.

[40] Wood SN, Goude Y, Shaw S (2015). Generalized additive models for large data sets. J. R. Stat. Soc. Ser. C Appl. Stat..

[41] Clark DP (1974). The influence of rainfall on the densities of adult *Chortoicetes terminifera* (Walker) in central western New South Wales, 1965–73. Aust. J. Zool..

[42] Shelford VE (1963). The ecology of North America. Ecol. N. Am..

[43] Deveson ED (2013). Satellite normalized difference vegetation index data used in managing Australian plague locusts. J. Appl. Remote Sens..

[44] Kuhnert PM, Lucchesi L (2018). Vizumap: An R package for visualizing uncertainty in spatial data.

[45] Lucchesi LR, Wikle CK (2017). Visualizing uncertainty in areal data with bivariate choropleth maps, map pixelation and glyph rotation. Stat.

[46] Benfekih L, Chara B, Doumandji-Mitiche B (2002). Influence of anthropogenic impact on the habitats and swarming risks of Dociostaurus maroccanus and Locusta migratoria (Orthoptera, Acrididae) in the Algerian Sahara and the semi-arid zone. J. Orthoptera Res..

[47] Štrumbelj E, Kononenko I (2014). Explaining prediction models and individual predictions with feature contributions. Knowl. Inf. Syst..

[48] Escorihuela MJ (2018). SMOS based high resolution soil moisture estimates for desert locust preventive management. Remote Sens. Appl. Soc. Environ..

[49] Myneni RB, Williams DL (1994). On the relationship between FAPAR and NDVI. Remote Sens. Environ..

[50] Hu G (2019). Long-term seasonal forecasting of a major migrant insect pest: the brown planthopper in the Lower Yangtze River Valley. J. Pest Sci..

