# Publisher Correction: Daily mapping of Australian Plague Locust abundance

**DOI:** 10.1038/s41598-020-79011-9

**Published:** 2020-12-15

**Authors:** Stéphane Mangeon, Allan Spessa, Edward Deveson, Ross Darnell, Darren J. Kriticos

**Affiliations:** 1 Commonwealth Scientifc and Industrial Research Organisation, Brisbane, Data61 Australia; 2 Australian Plague Locust Commission, 50 Collie St, Fyshwick, ACT 2609 Australia; 3 Fenner School of Environment and Society, Australian National University, Linnaeus Way, Acton, ACT 2601 Australia; 4 Commonwealth Scientifc and Industrial Research Organisation, Health and Biosecurity, Canberra, Australia; 5 School of Biological Sciences, University of Queensland, St. Lucia, QLD Australia

Correction to: *Scientific Reports* 10.1038/s41598-020-73897-1, published online 09 October 2020

In the original version of this Article, Allan Spessa was incorrectly affiliated with ‘Fenner School of Environment and Society, Australian National University, Linnaeus Way, Acton, ACT, 2601, Australia’. The correct affiliation is listed below.

Australian Plague Locust Commission, 50 Collie St, Fyshwick, ACT, 2609, Australia.

This error has now been corrected in the PDF and HTML versions of the Article, and in the accompanying Supplementary Information file.

