# Supplementary material for: Daily mapping of Australian Plague Locust abundance
Source: Sci Rep. 2020 Oct 9;10:16915. doi: 10.1038/s41598-020-73897-1 (PMC7547006; doi:10.1038/s41598-020-73897-1)
Supplement: Supplementary file 1 [file 41598_2020_73897_MOESM1_ESM.pdf]

Title: Daily mapping of Australian Plague Locust abundance

Stéphane Mangeon[1*], Allan Spessa[2,3], Edward Deveson[2,3], Ross Darnell[1] and Darren J. Kriticos[4,5]

[1] Commonwealth Scientific and Industrial Research Organisation, Data61, Brisbane, Australia
[2] Australian Plague Locust Commission, 50 Collie St, Fyshwick, ACT 2609, Australia
[3] Fenner School of Environment and Society, Australian National University, Linnaeus Way, Acton, ACT 2601, Australia
[4] Commonwealth Scientific and Industrial Research Organisation, Health and Biosecurity, Canberra, Australia
[5] School of Biological Sciences, University of Queensland, St. Lucia, Queensland, Australia

*Corresponding Author (stephane.mangeon@csiro.au)

Supplementary Material

Density classes to absolute density translation

Supplementary Table S1. Nymph and adult density in the APLC dataset and their corresponding population density, sampled from a normal distribution.

	Survey densities (classes)		Proportion of	Normal distribution (densities as count per m^2)	
**Stage**	**Name**	**Value**	**surveys (%)**	**Mean**	**Standard deviation**
**All**	Absence	0	34.08	0	N/A
**Nymph**	Present	1	5.48	3	1
**Nymph**	Numerous	2	1.57	18	6
**Nymph**	Sub-Band	3	0.39	55.5	12.25
**Nymph**	Band	4	0.94	140.5	29.75
**Adult**	Isolated	1	33.87	0.0105	0.04750
**Adult**	Scattered	2	16.46	0.051	0.0245
**Adult**	Numerous-Low	3	4.73	0.2	0.05
**Adult**	Numerous-High	4	2.48	0.4	0.05
**Adult**	Concentration	5	1.73	1.75	0.625
**Adult**	Low Density Swarm	6	0.88	6.5	1.75
**Adult**	Medium Density Swam	7	0.22	30	10
**Adult**	High Density Swarm	8	0.03	125	37.5

Our survey data contain 27 years of data, with an average of 9 000 records each year sampling from ~ 1 million km^2 of locust habitats. Not all habitat regions were sampled every month, and survey locations were not fixed, but frequently occur along repeated routes.

In the surveys, locust lifestage and abundance data are recorded during standard near-road foot transects of 1 x 250 m. Data are georeferenced by GPS. For further details on the survey protocol, see appendix in [28]. Sequential density classes represent a roughly geometric increase in population density, for this study we transform these density classes into a consistent metric system (individuals m^{-2}). Density classes were defined by a minimum and maximum density. In order to convert between the two, for each survey site, we sample the density from a normal distribution corresponding to each survey density class (Table S1). To do this, we take the mean of the distribution as the middle point between the minimum and maximum density for that class, meanwhile the standard deviation is set such that there are 2 standard deviations between the mean and the minimum/maximum. For example, for the first nymph density class, the minimum density was 1, and the maximum 5, thus we sample from a normal distribution with mean 3 and standard deviation 1. Hence, we transform discrete survey data into estimated continuous abundance data, the densities of nymphs and adults become comparable, and we can use regression algorithms with the dataset. Nevertheless, this introduces a ceiling to our model's performance, as this

sampling is random. A simple regression model between the mean and standard deviation (i.e. $\sigma = a * \mu + b$) finds that the standard deviation is about 30% of the mean, and a best performance of at most 70% that cannot be improved through our model. We chose to use a normal distribution to account for bias in surveyors' ability to distinguish between density categories. From a statistical standpoint, other distributions, such as uniform or truncated log-normal, could also have been valid candidates.

Supplementary Table S2. Ancillary variables which were highly correlated, with correlation coefficients above 0.9. These were used to remove variables from our analysis. For instance, high sand content strongly correlates to low clay content. And we can reduce a model's complexity by only including one of these.

Variable 1	Variable 2	Correlation Coefficient
Sand content (%)	Clay content (%)	-0.94
min. Temperature, 10 day mean	min. Temperature, 10 day min.	0.95
min. Temperature, 60 day mean	max. Temperature, 60 day mean	0.95
max. Temperature, 60 day mean	min. Temperature, 60 day max.	0.90
max. Temperature, 10 day mean	max. Temperature, 10 day min.	0.93
Sunlight, 60 day mean	Sunlight, 60 day max.	0.90
Daylength	Sunlight, 10 day max	0.94
Daily rain, 10 day max.	Daily rain, 10 day mean	0.93

Further Details on our Generalized Additive Models (GAM):

When fitting our GAMs, we use the negative binomial family, with a square-root link function. The formula used can be expressed as:

(Eq. S1) $\rho^{1/6} \sim S(longitude, latitude, day\ of\ year) + \sum_i^{(10\ or\ 27)} S(X_i)$

Where ρ is the density of locust, S denotes a smooth term (low-rank isotropic smoothers) which leverages thin plate regression splines (these are the default in the mgcv R library), and X_i is one of the covariates (10 or 27) we use in our GAM (see Table 1).

In the R computing language, this approach can be reproduced using the mgcv library through the following line of code:

```
gam.model <- bam(gam_formula, family=nb(theta=NULL,link="sqrt"), data = gam_df)
```

Where gam_formula can be written following Supplementary Material Equation S1, and gam_df is a data frame which contains the predictor (X_i as well as *longitude, latitude* and *day of year* in the same equation).

Note it may be useful to think of this equation when analysing Figure 1, S1, S2, and S3, which shows the form of the smooth function (S) relating the covariates (X) to the density of locust (ρ).

Estimating Uncertainty in Figure 2:

As we discussed previously in this Supplementary Material, we can estimate the uncertainty due to our random sampling to turn categories into densities using a simple linear model such that the uncertainty due to sampling ($\sigma_{sampling}$) can be expressed as a linear function of the predicted value (μ): $\sigma_{sampling} = a * \mu + b$. Meanwhile, our GAM models include uncertainty in their fit, which we can express as σ_{model}. As the sampling was done randomly, the two error terms are unrelated, thus, we can obtain the full uncertainty, as a standard deviation expressed as $\sigma = \sqrt{\sigma_{model}^2 + \sigma_{sampling}^2}$.

We acknowledge Supplementary Material Figure S4 can be difficult to interpret. For illustration purposes, take day 320 (the middle-top panel), the figure can be thought of as a spatial map of Australia (longitude as the x-axis and latitude as the y-

axis), which shows that around day 320 of the year (start of November) the southern regions would expect a higher *C. terminifera* abundance (yellow) than the northern regions. Importantly, this contribution could not be accounted for by our other covariates alone (temperature, rainfall etc…).

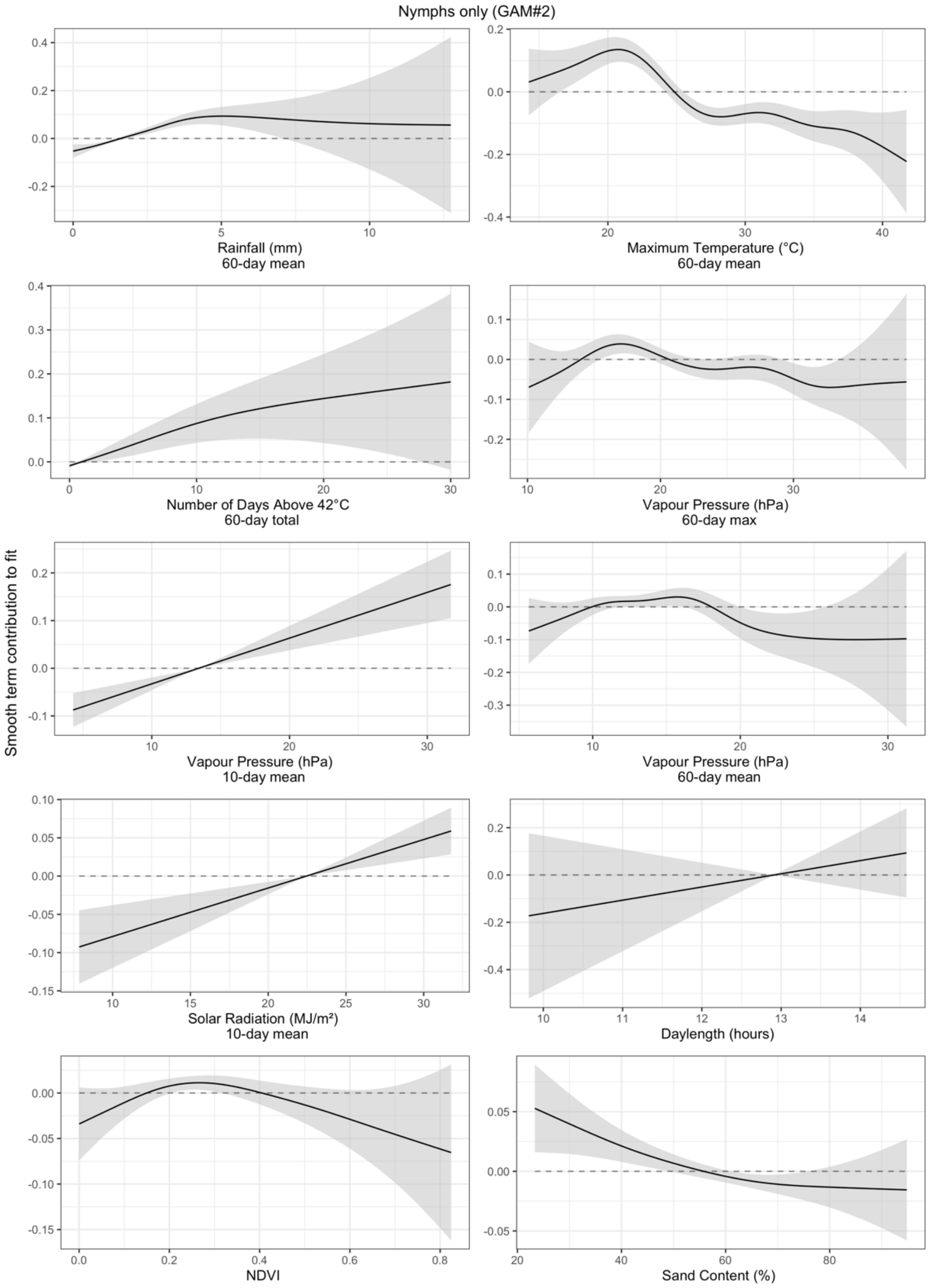

Supplementary Figure S1. The smooth term contribution to our fit of nymph density GAM for a reduced set of variables (GAM#2 in Table 2). The solid line shows the model fit, and the shaded area shows 2 standard deviations. Notwithstanding the influence of other covariates, these plots can be used to interpret the influence of each predictor on the abundance of locusts. A positive spline corresponds to higher abundance estimates, a negative one to lower estimates. Large shaded areas can be due to an uncertain relationship, or a lack of data.

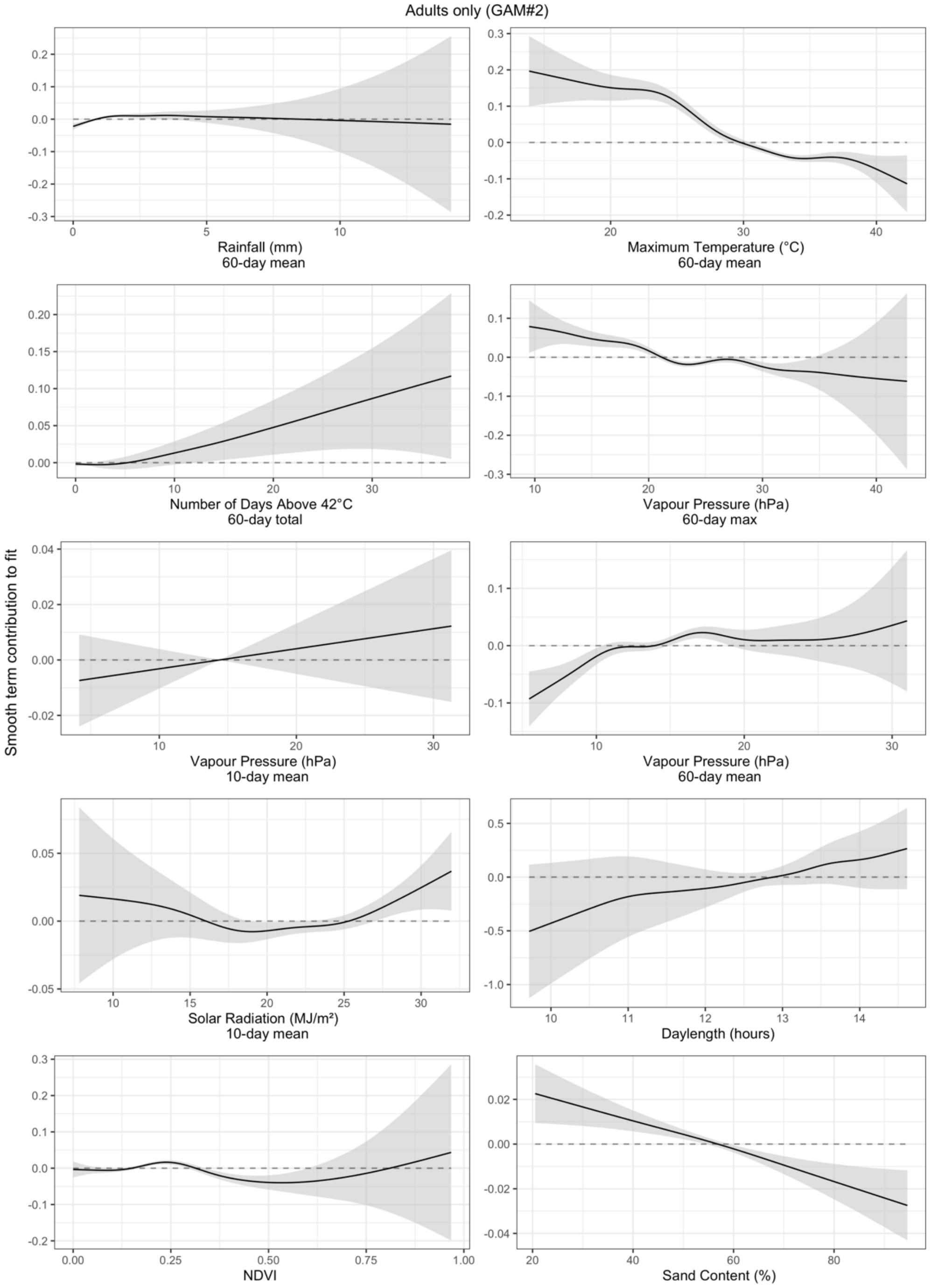

Supplementary Figure S2. The smooth term contribution to our fit of adult density GAM for a reduced set of variables (GAM#2 in Table 2). The solid line shows the model fit, and the shaded area shows 2 standard deviations. Notwithstanding the influence of other covariates, these plots can be used to interpret the influence of each predictor on the abundance of locusts. A positive spline corresponds to higher abundance estimates, a negative one to lower estimates. Large shaded areas can be due to an uncertain relationship, or a lack of data.

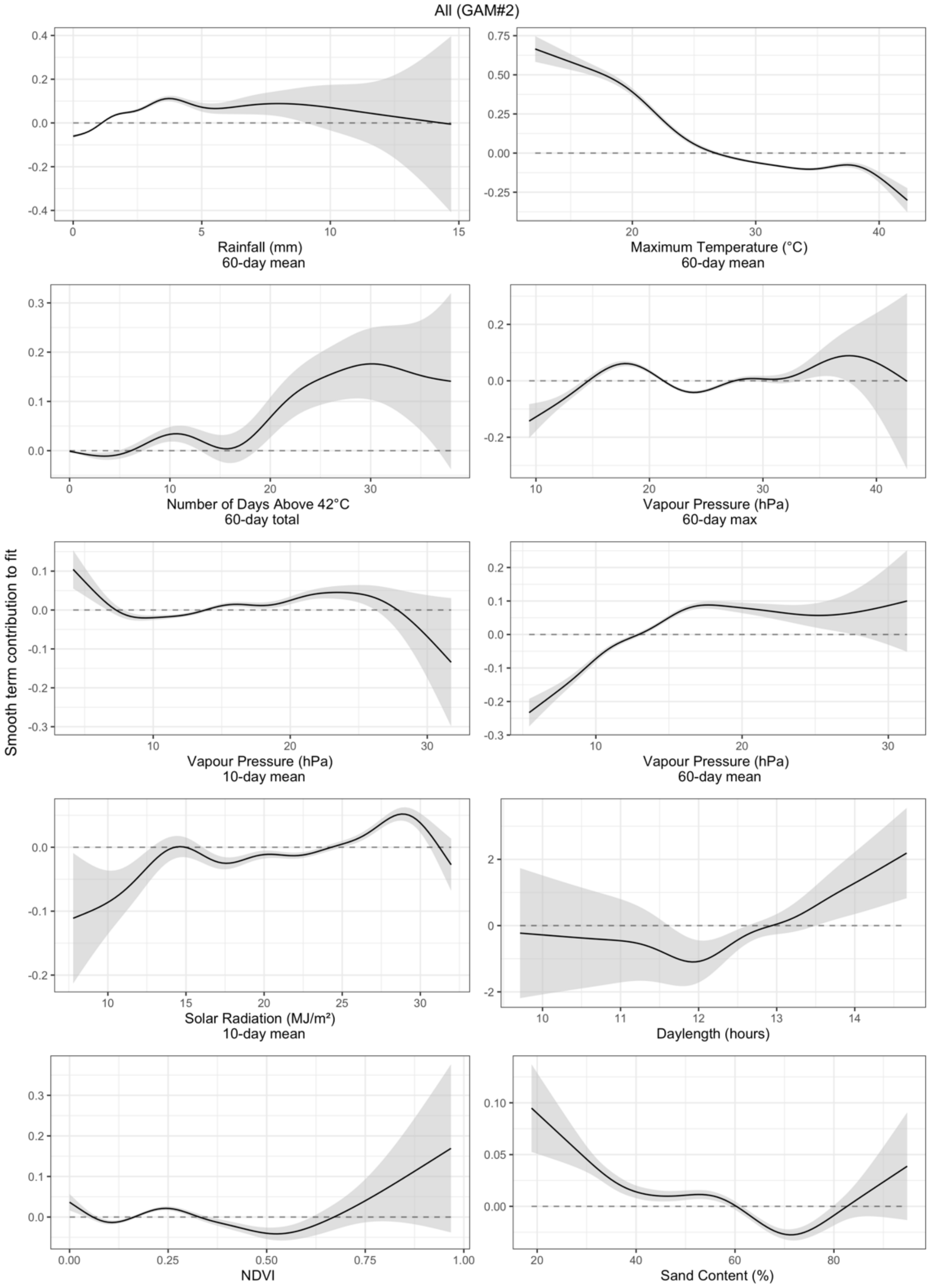

Supplementary Figure S3. The smooth term contribution to our fit of "all densities" GAM for a reduced set of variables (GAM#2 in Table 2). The solid line shows the model fit, and the shaded area shows 2 standard deviations. Notwithstanding the influence of other covariates, these plots can be used to interpret the influence of each covariate on the abundance of locusts. A positive spline corresponds to higher abundance estimates, a negative one to lower abundance. Large shaded areas can be due to an uncertain relationship, or a lack of data. This figure is the unedited version of Figure 1 in the main text, where we focused on areas with low uncertainty, and on 6 variables of particular interest.

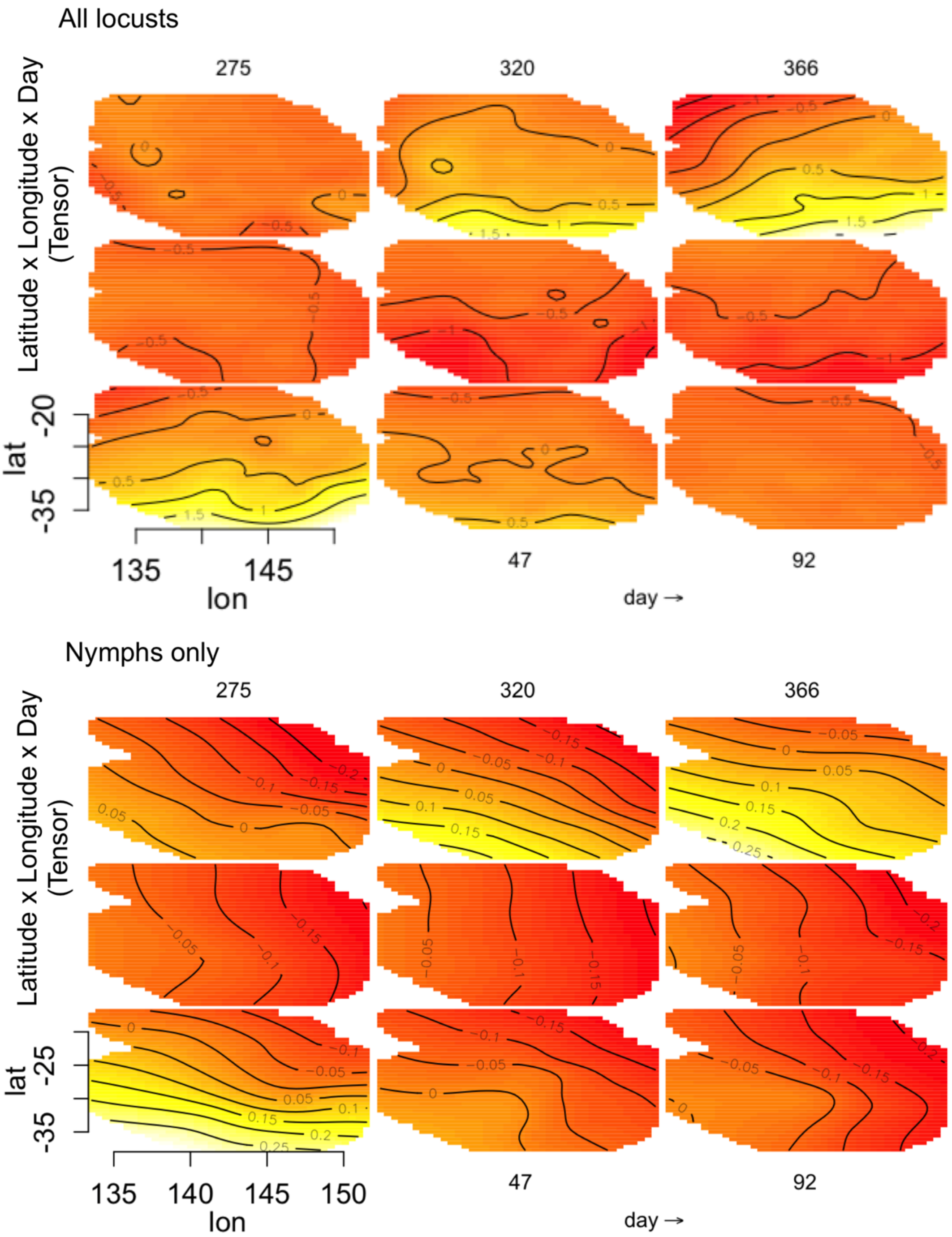

Supplementary Figure S4. The space-time tensor for all densities (top) and nymphs-only (bottom), under a reduced set of variables (GAM#2), the first term in Eq.S1: $S(longitude, latitude, day\ of\ year)$. The figure shows the contribution of the spatial and temporal location to the response (abundance) through contour lines and colours, from yellow (positive, leading to higher abundance), to red (negative, leading to lower abundance). Each facet shows a different day (from 1 for the first of January, to 366 for the following 1st of January), while the x and y axis show the longitude and latitude respectively.

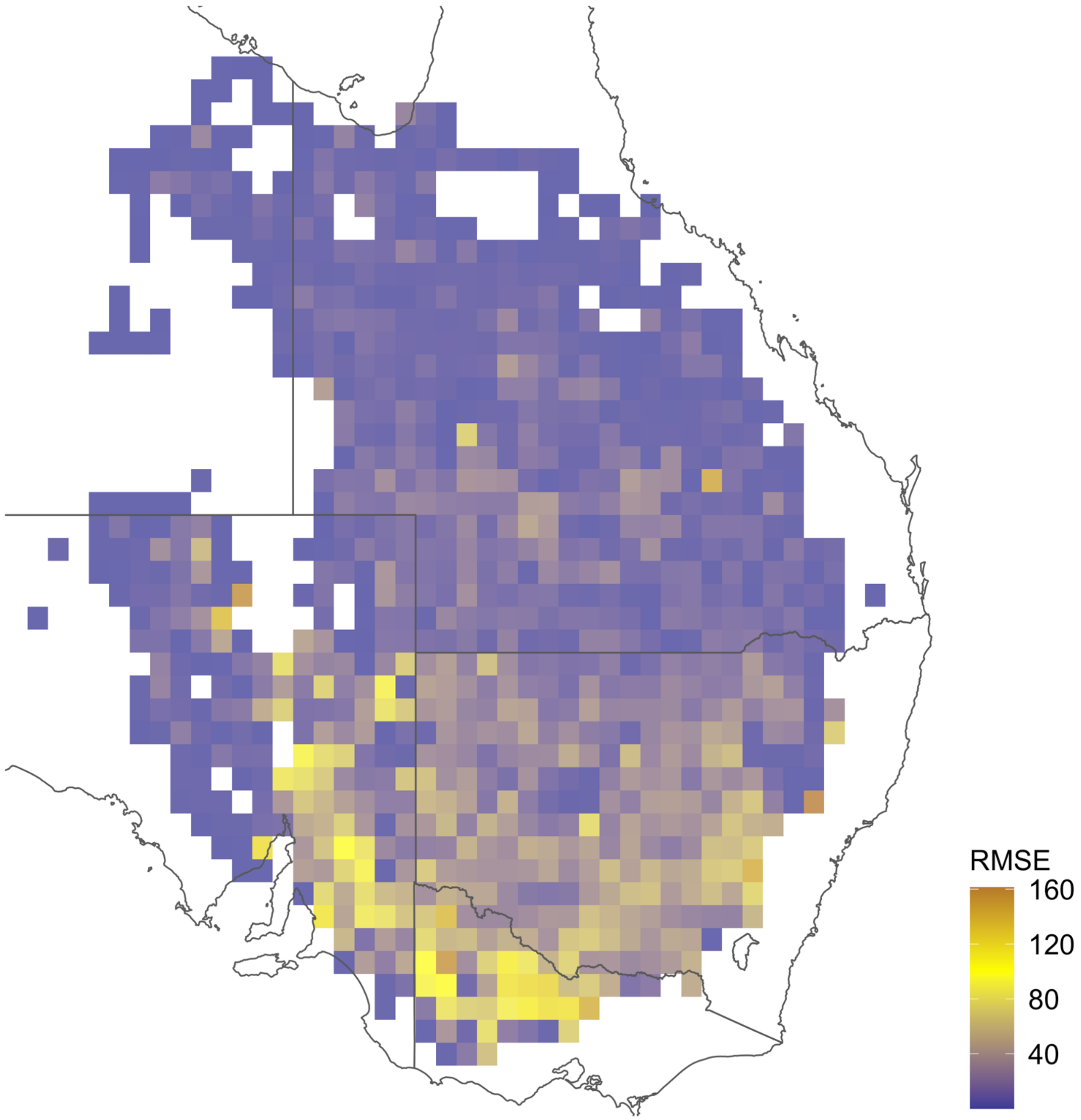

Supplementary Figure S5. The Root-Mean-Square Error (RMSE) of our estimates (compared with the surveyed locust density), gridded to 0.5x0.5 degrees to show its spatial patterns. Maps produced in R (v3.6.0) with the ggplot and ozmaps libraries (see https://cran.r-project.org/).

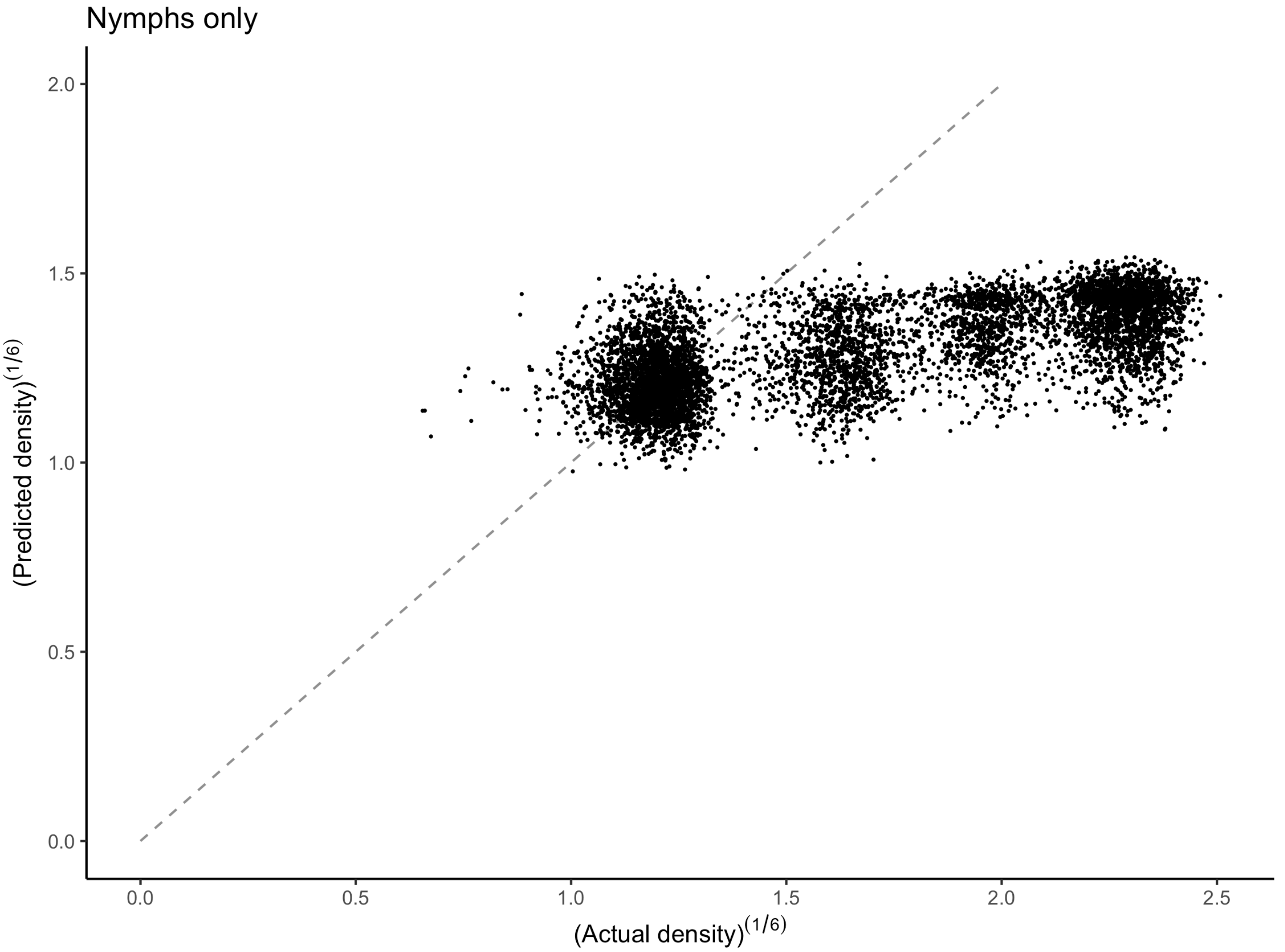

Supplementary Figure S6. A scatterplot showing observed vs predicted densities for our model of nymphs only. The dashed line shows the identity line (observed equals estimated). We can see the model underestimates the densities above 1.25. Due to the use of a 6th root transform, these effects drastically impact the estimate of high-densities.
